# Low incidence of venous thromboembolic complications following single-port robotic surgeries for upper and lower tract urological malignancies: a report from the Single-Port Advanced Research Consortium (SPARC)

**DOI:** 10.1007/s11701-025-02796-2

**Published:** 2025-10-25

**Authors:** Nicolas A. Soputro, Kennedy E. Okhawere, Yuzhi Wang, Michael E. Raver, Eugenio Bologna, Ruben Sauer Calvo, Elizabeth Snajdar, Narmina Khammandova, Jaya S. Chavali, Carter D. Mikesell, Adriana M. Pedraza, Indu Saini, Adam Lorentz, Bertram Yuh, Ryan J. Nelson, David I. Lee, Jean V. Joseph, Marcio C. Moschovas, Vipul Patel, Simone Crivellaro, Moses Kim, Jeffrey W. Nix, Riccardo Autorino, Mutahar Ahmed, Michael D. Stifelman, Craig Rogers, Ketan K. Badani, Jihad Kaouk

**Affiliations:** 1 Glickman Urological & Kidney Institute, Cleveland Clinic, 9500 Euclid Avenue, Cleveland, OH Q1044195 USA; 2 Mount Sinai Hospital, New York City, NY USA; 3 Henry Ford Hospital, Detroit, MI USA; 4 Hackensack Meridien School of Medicine, Hackensack, NJ USA; 5 RUSH University Medical Center, Chicago, IL USA; 6 University of Illinois at Chicago (UIC) Health, Chicago, IL USA; 7 Michigan Institute of Urology, Detroit, MI USA; 8 University of California Irvine, Orange, CA USA; 9 Emory University Hospital, Atlanta, GA USA; 10 City of Hope, Duarte, CA USA; 11 University of Rochester Medical Center, Rochester, NY USA; 12 Advent Health, Celebration, FL USA; 13 Orange County Urology Associates, Laguna Hills, CA USA; 14 University of Alabama at Birmingham (UAB), Birmingham, AL USA; 15 Hackensack Meridien Health, Hackensack, NJ USA

**Keywords:** Robotic surgery, Prostate cancer, Kidney cancer, Single port, Deep venous thrombosis, Pulmonary embolism

## Abstract

With the increasing utility of the single-port (SP) robotic platform, there remains limited evidence surrounding the perioperative morbidity associated with the novel techniques. This study sought to evaluate the incidence of venous thromboembolic events (VTE) associated with the SP procedures for upper and lower tract urological malignancies, based on the multi-institutional experience of the Single-Port Advanced Research Consortium (SPARC), involving a total of 2286 patients. The group comprised 1886 (82.5%) and 400 (17.5%) cases of SP robotic radical prostatectomy (RARP) and SP robotic partial nephrectomy (RAPN) that were completed between 2018 and 2024. In addition to the perioperative variables, all patients were stratified into low, moderate, and high risk for VTE according to the Caprini scoring system. Notably, no cases of VTE were identified following SP-RAPN, while eight cases of DVT (0.42%) and two cases of PE (0.11%) were diagnosed following SP-RARP. Appreciating the different risk factors of VTE, a higher Caprini score was identified to be associated with VTE, with every one-point increase in the score contributing to 1.6 times higher odds of VTE following SP-RARP. In summary, this study demonstrated the relatively low incidence of VTE associated with SP robotic procedures for upper and lower tract urological malignancies (0.44%). The utility of validated tools such as the Caprini scoring system can provide additional benefits to identify at-risk patients and provide foundations for further research to better select candidates for mechanical and pharmacological thromboprophylaxis and, thus, ensure satisfactory perioperative outcomes.

**Supplementary Information:**

The online version contains supplementary material available at 10.1007/s11701-025-02796-2.

## Introduction

The introduction of robotic surgery in the early 2000s has opened a new frontier within the domains of minimally invasive urological surgery, particularly for the management of various urological malignancies, such as prostate and kidney cancer. When compared to the open and laparoscopic approaches, the robotic techniques have been previously shown to confer additional benefits in promoting better postoperative recovery and reducing perioperative morbidity [1, 2]. With the continuing advances in surgical technologies and techniques, recent years have witnessed the introduction of a novel purpose-built single-port (SP) robotic platform with unique features, which included the narrow profile of the single robotic arm that can simultaneously accommodate one flexible high-definition 3D camera and three double-jointed robotic instruments [3]. Compared to the traditional multi-port robotic surgical platforms, these distinctive features of the SP promise improved maneuverability and ergonomics in smaller and shallow surgical working spaces, which provide an additional opportunity for surgeons to consider less invasive and more regionalized surgical approaches that only pertain to the relevant anatomy [4, 5].

Following Food and Drug Administration (FDA) approval in 2018 [6], the safety and feasibility of the SP robotic platform for various upper tract and lower tract urological procedures have been demonstrated, including for the different approaches of robotic radical prostatectomy (RARP) and robotic partial nephrectomy (RAPN) [4]. In addition, recent studies have also showcased the additional benefits of the more regionalized SP techniques, especially as they promote further enhancement in postoperative recovery favoring opioid-sparing outpatient procedures as well as facilitating an expanded clinical indication to also include patients with multiple previous abdominal surgeries or hostile abdomen [7, 8]. With the increasing adoption of the SP platform across the USA, Asia, and Europe, a better understanding of the different clinical benefits and the perioperative morbidity profile associated with SP procedures remains paramount. Among the different postoperative complications, it is important to consider the risk of serious complications, such as venous thromboembolism (VTE) and bleeding, especially when considering the surgical management of urological malignancies. Hence, the present study aimed to evaluate the incidence of VTE associated with SP upper and lower tract robotic urological procedures for the management of urological cancers.

## Materials and methods

A retrospective review was performed on the prospectively maintained, institution review board (IRB)-approved multi-institutional database of the Single Port Advanced Research Consortium (SPARC) to investigate the incidence of VTE both in the postoperative inpatient and outpatient settings, including but not limited to deep venous thrombosis (DVT) and pulmonary embolism (PE), following SP-RARP and SP-RAPN that were performed between October 2018 and March 2024 at 14 different institutions. The two procedures were selected as they represent the most common SP robotic procedures performed for upper and lower tract urological malignancies, respectively.

Following its inception in 2018, SPARC has grown to become the largest collaborative initiative involving 25 institutions across the USA that practice SP urological surgeries using the purpose-built SP robotic platform (Intuitive Surgical, Sunnyvale, CA). All de-identified clinical information was collected prospectively and stored in a Health Insurance Portability and Accountability Act (HIPAA)-compliant Research Electronic Data Capture (REDCap) database housed by the host institution. Individual data user agreements (DUA) between the host institution and each of the member institutions were obtained as part of the enrollment to the consortium and before any data sharing.

For this study, data were collected from 14 different institutions, with variables included baseline clinicodemographic characteristics, such as age, gender, ethnicity, preoperative laboratory workup, imaging, and biopsy results. Baseline comorbidities were collected in accordance with the Charlson Comorbidity Index (CCI) with a strong emphasis on the previous history of VTE. The preoperative risk of VTE was calculated using the Caprini score, which was then used to stratify patients into low (0–2), moderate (3–4), or high-risk (≥ 5) for postoperative VTE complications [9]. Intraoperative parameters included the specific surgical approaches, total operating time, estimated intraoperative blood loss (EBL), as well as evidence of any need for additional port, conversion, or intraoperative complications. Postoperative variables comprised the total inpatient length of stay, analgesia usage, Foley urinary catheter duration, as well as the 90-day incidence of postoperative complication and hospital readmission. Postoperative complications were reported in accordance with the Clavien–Dindo classification, with major complications defined as those scoring ≥ 3a [10].

The various surgical approaches for SP-RARP have been described previously, which included transperitoneal, extraperitoneal, transperineal, transvesical, and retzius-sparing access [11–15]. In this present series, only transperitoneal, extraperitoneal, and transvesical SP-RARP were included in our analysis. All three procedures were routinely performed through a midline surgical incision, through which the purpose-built SP Access Kit (Intuitive Surgical, Sunnyvale, CA) was secured to facilitate robot docking via a floating-docking technique [16]. In addition to the multichannel cannula for the flexible endoscopic camera and the double-jointed SP instruments, the SP Access Kit also houses a 12 mm assistant port adjacent to the multichannel cannula that is commonly used for the remotely operated suction irrigation (ROSI) system (Vascular Technology Inc. [VTI], Nashua, NH) as well as an additional port on the side of the bubble chamber that is routinely used for the placement of insufflation trocar. Of note, while the surgical approaches for transperitoneal and extraperitoneal SP-RARP have similarities to the previously described techniques with the MP robotic platform, the SP transvesical approach remains unique given the direct percutaneous access into the bladder from a midline suprapubic incision and the ability to complete all dissection and vesicourethral anastomosis (VUA) steps from within the confines of the bladder under a low pneumovesical pressure [14, 17].

Similar to SP-RARP, recent years have witnessed the introduction of various surgical approaches of SP-RAPN with the procedure now able to be completed via either a transperitoneal or retroperitoneal approach, as previously described in the literature [5, 18]. The placement of an additional port or use of a surgical drain tube is typically not indicated, but such decision remains at the discretion of the surgeon.

With regard to VTE prophylaxis, our institutions routinely administers 5000 units of heparin preoperatively, unless otherwise contraindicated. In addition, patients are routinely placed on bilateral lower extremity pneumatic compression devices intraoperatively as a standard VTE prophylaxis. The preference of postoperative prophylaxis with either 5000 units of subcutaneous heparin every 8–12 h or 40 mg of enoxaparin every 12–24 h remains dependent on the individual surgical teams as well as the specific patient factors, such as their BMI, previous history of VTE, as well as underlying cardiopulmonary comorbidities. At the time of discharge, all patients with high-risk prostate cancer who underwent extraperitoneal SP-RARP with pelvic lymph node dissections are routinely prescribed 40 mg of daily subcutaneous enoxaparin for 21 days as per our institutional protocol.

Statistical analysis was performed using RStudio (R Packages for Statistical Computing, Vienna, Austria) with descriptive statistics as presented. Categorical variables were reported as the absolute and relative percent frequencies, while continuous variables were presented as the median and interquartile range (IQR). Logistic regression analysis was performed to evaluate the potential predictors of VTE, with variables with *p*-values of < 0.05 considered statistically significant.

## Results

A total of 2286 patients were included in our analysis, which comprised 1886 (82.5%) and 400 (17.5%) who underwent SP-RARP and SP-RAPN, respectively.

### Incidence of VTE following SP-RARP

The SP-RARP cohort had a median age of 64 years (IQR 59–69 years) and a median BMI of 28 kg/m^2^ (IQR 25.4–31.1 kg/m^2^), with 1225 (78.4%) noted to have a BMI higher than 25 kg/m^2^. As summarized in Table 1, the most common surgical indication for SP-RARP was intermediate-risk prostate cancer (*n* = 1058, 65.6%), followed by high- to very high-risk disease (*n* = 286, 17.7%) and very low- to low-risk disease (*n* = 268, 16.6%). Intraoperatively, the extraperitoneal approach represented the most common SP-RARP technique (*n* = 744, 45.6%), followed by the SP transperitoneal (*n* = 569, 34.9%) and transvesical (*n* = 319, 19.5%) approaches. The median total operative time for SP-RARP was 194 min (IQR 150–244 min), which included the completion of nerve-sparing and lymph node dissection (LND) in 86.8% and 66.4% of the cases, respectively. When evaluating the perioperative VTE risk based on the baseline comorbidities, our SP-RARP cohort had a median Caprini score of 5 (IQR 4–5) with 59.7% being classified as high risk, while the remaining was categorized as moderate risk for postoperative VTE.

**Table 1:** Baseline clinicodemographic variables and perioperative characteristics of all SP-RARP patients included in our analysis

	*n* = 1886
Baseline clinicodemographic variables	
Age (years), median (IQR)	64 (59–69)
BMI (kg/m^2^), median (IQR)	28 (25.4–31.1)
ASA Score	
ASA 1–2, *n* (%)	656 (34.8%)
ASA 3–4, *n* (%)	1230 (65.2%)
CCI score, median (IQR)	4 (3–5)
Previous abdominal surgery, *n* (%)	650 (34.5%)
Caprini score, median (IQR)	5 (4–5)
Low risk, *n* (%)	0 (0%)
Moderate risk, *n* (%)	760 (40.3%)
High risk, *n* (%)	1126 (59.7%)
NCCN prostate cancer risk categories	
Very low–low, *n* (%)	314 (16.6%)
Intermediate, *n* (%)	1237 (65.6%)
High–very high, *n* (%)	335 (17.7%)
Intraoperative parameters	
Surgical approach	
SP transperitoneal, *n* (%)	569 (34.9%)
SP extraperitoneal, *n* (%)	744 (45.6%)
SP transvesical, *n* (%)	319 (19.5%)
Total operating time (min), median (IQR)	194 (150–244)
Estimated blood loss (mL), median (IQR)	100 (50–100)
Nerve-sparing procedure, *n* (%)	1311 (86.8%)
Lymph node dissection, *n* (%)	1252 (66.4%)
Lymph node yield, median (IQR)	6 (4–10)
Intraoperative complication, *n* (%)	11 (0.8%)
Histopathological outcomes	
Specimen weight (grams), median (IQR)	47 (38–60)
Radical prostatectomy Gleason Grade Group	
Grade Group 1, *n* (%)	86 (5.4%)
Grade Group 2, *n* (%)	940 (59.6%)
Grade Group 3, *n* (%)	370 (23.4%)
Grade Group 4, *n* (%)	58 (3.7%)
Grade Group 5, *n* (%)	124 (7.9%)
Negative surgical margin, *n* (%)	1430 (75.8%)
Pathology T (pT) stage	
pT2, *n* (%)	951 (59.2%)
pT3a, *n* (%)	482 (30%)
pT3b, *n* (%)	168 (10.5%)
pT4, *n* (%)	5 (0.3%)
Pathology N (pN) stage	
pNx, *n* (%)	592 (36%)
pN0, *n* (%)	980 (59.6%)
pN1, *n* (%)	73 (4.4%)
Postoperative outcomes	
Length of stay (days), median (IQR)	0.7 (0.2–1)
Foley catheter duration (days), median (IQR)	7 (5–8)
90-day postoperative complication, *n* (%)	199 (10.6%)
90-day major postoperative complication, *n* (%)	67 (3.6%)

IQR, interquartile range; BMI, body mass index; ASA, American Society of Anesthesiologist; CCI, Charlson Comorbidity Index; NCCN, National Comprehensive Cancer Network; SP, single port

Within the SP-RARP cohort, VTE was identified in ten (0.53%) patients at a median of 7 days (IQR 5–8 days) following the respective surgery, with all cases confirmed on radiological images. Among the different types of postoperative complications, VTE represents 5% of the overall 90-day postoperative complications, with all being categorized as minor complications of Clavien–Dindo grade 2. When evaluated based on their Caprini scores, all the VTE cases were classified as high risk. The ten cases of VTE included eight (0.4%) cases of DVT and two (0.1%) cases of PE. The two cases of PE pertained to small subsegmental PEs that were diagnosed following extraperitoneal SP-RARP, one on a 76-year-old with a pT2N0 Grade Group 2 prostate cancer, while the other on a 57-year-old with a pT3aN1 Grade Group 2 prostate cancer, both with negative surgical margins. Given the acuity of the diagnosis timeline during the inpatient course, both patients were managed conservatively with early ambulation as well as both mechanical and pharmacological VTE prophylaxis until they were discharged home without any further clinical sequelae.

Of the eight cases of lower extremity DVT, six were identified following extraperitoneal SP-RARP, all of whom were noted to have pT2N0 Grade Group 2 prostate cancer. The sole cases of DVT that were diagnosed following transperitoneal and transvesical SP-RARP pertained to a 69-year-old with a pT2N0 Grade Group 2 prostate cancer and a 56-year-old with a pT2Nx Grade Group 5 prostate cancer, respectively. In terms of risk factors, while none of the patients had any previous history of VTE, all eight patients were considered overweight with a median BMI of 28.2 kg/m^2^ (IQR 25.6–31.3 kg/m^2^). All cases were diagnosed and confirmed on lower limb ultrasonography within 7–8 days following the surgery with the earliest presentation occurring on postoperative day (POD) 3. All cases were managed with therapeutic anticoagulation without any further complications.

**Fig. 1:**
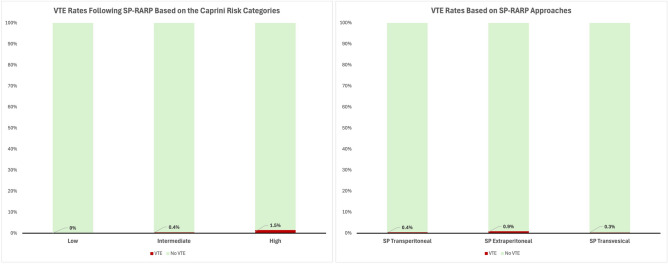
The different rates of postoperative VTE according the Caprini risk categories and SP-RARP approaches

Given the diagnosis of VTE in our SP-RARP cohort, we performed an additional statistical analysis to evaluate the potential clinicodemographic predictors of VTE. Of the different univariate analyses, only higher Caprini scores were identified as a significant predictor of VTE (odds ratio [OR] 1.664; 95% confidence interval [CI] 1.159–2.389, *p* = 0.006). Other perioperative variables, such as age (OR 1.054; 95% CI 0.096–1.156, *p* = 0.266), BMI (OR 1.054; 95% CI 0.090–1.155, *p* = 0.725), previous history of VTE (OR 1.540; 95% CI 0.803–2.956, *p* = 0.194), baseline prostate cancer disease characteristics, as well as intraoperative factors including the total operating time (OR 1.003; 95% CI 0.998–1.007, *p* = 0.243), EBL (OR 1.001; 95% CI 0.998–1.003, *p* = 0.412), LND (OR 2.033; 95%CI 0.430–9.606, *p* = 0.371), and the final histopathological outcomes were not shown to contribute to increased risk of VTE. The different rates of VTE according to the Caprini risk categories are also summarized in Fig. 1.

## Incidence of VTE following SP-RAPN

A total of 400 consecutive cases of SP-RAPN were included in this present study. The group consisted of 214 (53.5%) males with a median age of 60 years (IQR 52–67 years) and a median BMI of 28.8 kg/m^2^ (IQR 25.4–32.9 kg/m^2^). Compared to the SP-RARP cohort, the SP-RAPN group had a relatively reduced VTE risk with a median Caprini score of 4 (IQR 4–5), especially with a majority of the patients being classified as moderate risk (*n* = 194, 56.7%). The baseline clinical characteristics of our patients were summarized in Table 2.

**Table 2:** Baseline clinicodemographic variables and perioperative characteristics of all SP-RAPN patients included in our analysis

	*n* = 400
Baseline clinicodemographic variables	
Age (years), median (IQR)	60 (52–67)
BMI (kg/m^2^), median (IQR)	28.8 (25.4–32.9)
Gender (male), *n* (%)	214 (53.5%)
ASA score	
ASA 1–2, *n* (%)	163 (47.4%)
ASA 3–4, *n* (%)	181 (52.6%)
CCI score, median (IQR)	3 (2–4)
Previous abdominal surgery, *n* (%)	166 (50.4%)
Caprini score, median (IQR)	4 (4–5)
Low risk, *n* (%)	8 (2.3%)
Moderate risk, *n* (%)	194 (56.7%)
High risk, *n* (%)	140 (40.9%)
Baseline glomerular filtration rate (mL/min/1.73m^2^), median (IQR)	84.9 (66.7–98.1)
Laterality (right), *n* (%)	189 (59.6%)
Largest tumor diameter (cm), median (IQR)	2.7 (2–3.8)
RENAL nephrometry score, median (IQR)	6 (5–7)
Intraoperative parameters	
Total operating time (min), median (IQR)	144 (109.5–194.5)
Warm ischemia, *n* (%)	263 (89.8%)
Ischemia time (minutes), median (IQR)	18 (13–25)
Estimated blood loss (mL), median (IQR)	50 (30–100)
Intraoperative complication, *n* (%)	7 (1.8%)
Histopathological outcomes	
Malignant histology, *n* (%)	220 (78%)
Clear cell RCC, *n* (%)	153 (69.5%)
Papillary RCC, *n* (%)	41 (18.6%)
Chromophobe RCC, *n* (%)	15 (6.8%)
Negative surgical margin, *n* (%)	281 (92.1%)
Pathology T (pT) stage	
pT1a, *n* (%)	191 (74.3%)
pT1b, *n* (%)	41 (16%)
pT2a, *n* (%)	5 (1.9%)
pT2b, *n* (%)	2 (0.8%)
pT3a, *n* (%)	17 (6.6%)
pT4, *n* (%)	1 (0.4%)
Postoperative outcomes	
Length of stay (days), median (IQR)	1 (1–1)
Postoperative glomerular filtration rate (mL/min/1.73m^2^), median (IQR)	76.8 (61.6–93.9)
90-day postoperative complication, *n* (%)	22 (5.5%)
90-day major postoperative complication, *n* (%)	8 (2%)

With regard to the perioperative outcomes, the median total operating time was 144 min (IQR 110–195 min). Most of the procedures (*n* = 263, 89.8%) were completed under warm ischemia with a median warm ischemia time of 18 min (IQR 13–25 min). Malignant histology was identified in 220 (78%) cases, most of which pertained to clear cell renal cell carcinoma (RCC) (*n* = 153, 69.5%). In terms of postoperative morbidity, 90-day postoperative complication was noted in 22 (5.5%) patients, none of which were related to VTE.

## Discussion

To our knowledge, this study represents the first to report the incidence of VTE following SP robotic surgeries for upper and lower tract urological malignancies. Based on our multi-institutional experience involving 2286 patients, we have identified a relatively low incidence of VTE following SP urological procedures (0.44%), none of which were attributed to SP-RAPN. When compared to the literature as summarized in Table 3, the 0.53% and 0% incidence of VTE associated with SP-RARP and SP-RAPN in our cohort appeared to be lower compared to the previously reported 0.6–5.7% and 0.4–1.7% risks of VTE for MP-RARP and MP-RAPN, respectively [19–37].

**Table 3:** Literature review summarizing the incidence of VTE following minimally invasive radical prostatectomy and partial nephrectomy

Authors (year)	Country	Approach	*n*	Incidence
Partial nephrectomy				
Harris et al*.* (2019) [19]	USA	MIS	12,018	DVT = 1.2% PE = 0.5%
Schmid et al*.* (2016) [20]	USA	MIS	692	DVT/PE = 1.4%
Autorino et al*.* (2015) [21]	USA	MIS	2210	PE = 0.32%
Larson et al*.* (2015) [22]	USA	Robotic	1532	VTE = 0.46%
Ting et al*.* (2015) [23]	Australia	Robotic	77	VTE = 1.3%
Zeccolini et al*.* (2015) [24]	Italy	Robotic	60	PE = 1.7%
Alberts et al*.* (2014) [25]	USA	MIS	1690	DVT = 0.4% PE = 0.4% VTE = 0.7%
Radical prostatectomy				
Homer et al*.* (2024) [26]	USA	Robotic	11,811	PE = 0.6%
Meguro et al*.* (2022) [27]	Japan	Robotic	209	VTE = 5.7%
Tang et al*.* (2021) [28]	China	Laparoscopic	30	DVT = 23.3% PE = 0.2%
Frankel et al*.* (2020) [29]	USA	Robotic	3719	DVT = 0.91% PE = 0.73% VTE = 1.4%
Patel et al*.* (2020) [30]	USA	Robotic	187	VTE = 0.6%
Shi et al*.* (2018) [31]	China	Laparoscopic	205	DVT = 9.4% PE = 2.4%
Satkunasivam et al*.* (2016) [32]	USA	MIS	27,868	DVT = 0.6% PE = 0.5% VTE = 1%
Schmid et al*.* (2016) [20]	USA	MIS	4224	DVT = 32.4% PE = 13.6%
Abel et al*.* (2014) [33]	USA	Robotic	549	DVT = 1.3% PE = 0.55% VTE = 1.8%
Alberts et al*.* (2014) [25]	USA	MIS	12,865	DVT = 0.7% PE = 0.5% VTE = 1%
Benyo et al*.* (2014) [34]	Hungary	Laparoscopic	212	VTE = 1.4%
Saily et al*.* (2014) [35]	Finland	Robotic	398	DVT = 0.3% VTE = 0.25%
Liss et al*.* (2013) [36]	USA	Robotic	1000	DVT = 0.6% PE = 0.6%
Van Hemelrijck et al*.* (2013) [37]	Sweden	Laparoscopic with PLND	460	DVT = 1.8% PE = 0.92%
		Laparoscopic without PLND	4707	DVT = 0.51% PE = 0.18%

MIS, Minimally invasive surgery; DVT, deep venous thrombosis; PE, pulmonary embolism; VTE, venous thromboprophylaxis; PLND, pelvic lymph node dissection

When considering the different risk factors for VTE, the American College of Chest Physicians (ACCP) recommended the use of validated risk assessment tools, such as the Caprini and Rogers scores to stratify patients into either the ‘very low’, ‘low’, ‘moderate’, and ‘high’ risk categories [38]. The Caprini score was first introduced in 2005 and later modified in 2013 to include 35 different variables ranging from the baseline demographic characteristics, medical history including for active malignancy and previous history of DVT or PE, as well as intraoperative variables particularly the length of the procedure [9]. According to the ACCP guidelines, mechanical thromboprophylaxis, such as graduated compression stockings or pneumatic calf compressors, is recommended for patients in the low-risk category. The addition of pharmacological thromboprophylaxis, such as with either low molecular weight heparin (LMWH) or unfractionated heparin (UFH), remains important to consider in patients in moderate- and high-risk categories, especially in those undergoing cancer-related surgeries [38].

After applying the Caprini scoring system, we identified that most of the VTE cases in our series were diagnosed in patients who were considered high risk. Based on our logistic regression analysis, a higher Caprini score was shown to be associated with postoperative VTE, especially with every 1-unit increase in the Caprini score translating to 1.6 times higher odds of VTE. Despite this finding, there remain some limitations pertaining to the Caprini scoring system, especially within the domains of minimally invasive surgery for urological cancers. Of note, all the SP-RARP and SP-RAPN cases were considered major surgery (≥ 45 min) and automatically scored two points without any appreciation of the technical nuances of the procedures, such as LND and the differences between upper and lower tract procedures. Notably, RARP often requires a steep Trendelenburg positioning and pneumoperitoneum, which collectively may increase the risk of venous stasis, especially when compared with RAPN, which involves less pelvic manipulation [9].

The influence of PLND on the VTE risk associated with RARP has been previously demonstrated in the literature, especially with Patel et al*.* noting all their VTE cases to be identified in patients who underwent PLND and with Homer et al*.* appreciating the association between higher lymph node yield and increased risk of VTE [26, 30, 39]. Based on our experience, nine of the ten VTE cases were diagnosed in patients who had SP-RARP with PLND. Despite the lack of statistical significance, we also identified a positive correlation between the completion of PLND during SP-RARP and the increased risk of VTE (OR 2.033; 95%CI 0.430 – 9.606, *p* = 0.371). Although the pathophysiology remains unclear and with the need for further research to determine causality, it was postulated that the increased risk of VTE following PLND and during pelvic surgeries might relate to the compression of the pelvic veins and disruptions in the venous blood flow from the lower limb, which, when combined with the hypercoagulable state from the underlying malignancy, may hypothetically increase the predisposition to thrombus formation [30, 38].

While the findings in this study were important in providing insights into the risk and predictors of VTE associated with the novel SP robotic surgical approaches, this study is not without its limitations, with the first relating to its retrospective nature based on patients with symptomatic postoperative VTE presentations. Secondly, despite the relatively large multi-institutional cohort, the absence of VTE, such as in our population of SP-RAPN, did not necessarily exclude the risk of VTE in the population. In addition, the multi-institutional data captured in this study highlighted some heterogeneities in practice patterns, which may contribute to differences in the incidence of VTE. Notably, the introduction of the SP transvesical approach has provided an alternative to the standard template LND by limiting the dissection to the obturator lymph nodes. While this can reduce the morbidity associated with the standard LND, further studies remain necessary to understand the long-term oncological outcomes of this technique. Furthermore, our multi-institutional registry also did not capture the details surrounding the VTE prophylaxis protocol adopted by the individual surgeons and institutions.

When considering the utility of existing scoring systems, it is important to acknowledge that the Caprini score was developed for general surgical patients and not specific for patients with prostate or kidney cancers [39]. Although previous studies have identified the lack of benefits of pharmacological thromboprophylaxis in reducing the risk of VTE [30, 40, 41], it is important to appreciate that most of the studies did not specifically isolate patients with high Caprini scores and those who underwent LND, which may serve as the basis for future studies.

## Conclusion

In this study, we have demonstrated the low incidence of VTE associated with SP robotic procedures (0.44%) for upper and lower tract urological malignancies. While we did not identify any cases of VTE following SP-RAPN, the marginal risk of VTE associated with SP-RARP (0.53%) remained relatively lower compared to previous reports on MP-RARP. With the expanding repertoire of minimally invasive surgical techniques, a better appreciation of the perioperative morbidity profile associated with the novel approaches and more accurate identification of at-risk patients remains crucial to ensure the continuing favorable outcomes of our urological cancer procedures.

## Supplementary Information

Below is the link to the electronic supplementary material.

Supplementary file1 (PNG 153 KB) Fig. S1. Case contributions from participating institutions of the Single Port Advanced Research Consortium

## Data Availability

No datasets were generated or analyzed during the current study.
