# Supplementary figures and images for: Low incidence of venous thromboembolic complications following single-port robotic surgeries for upper and lower tract urological malignancies: a report from the Single-Port Advanced Research Consortium (SPARC)
Source: J Robot Surg. 2025 Oct 25;19(1):709. doi: 10.1007/s11701-025-02796-2 (PMC12553562; doi:10.1007/s11701-025-02796-2)

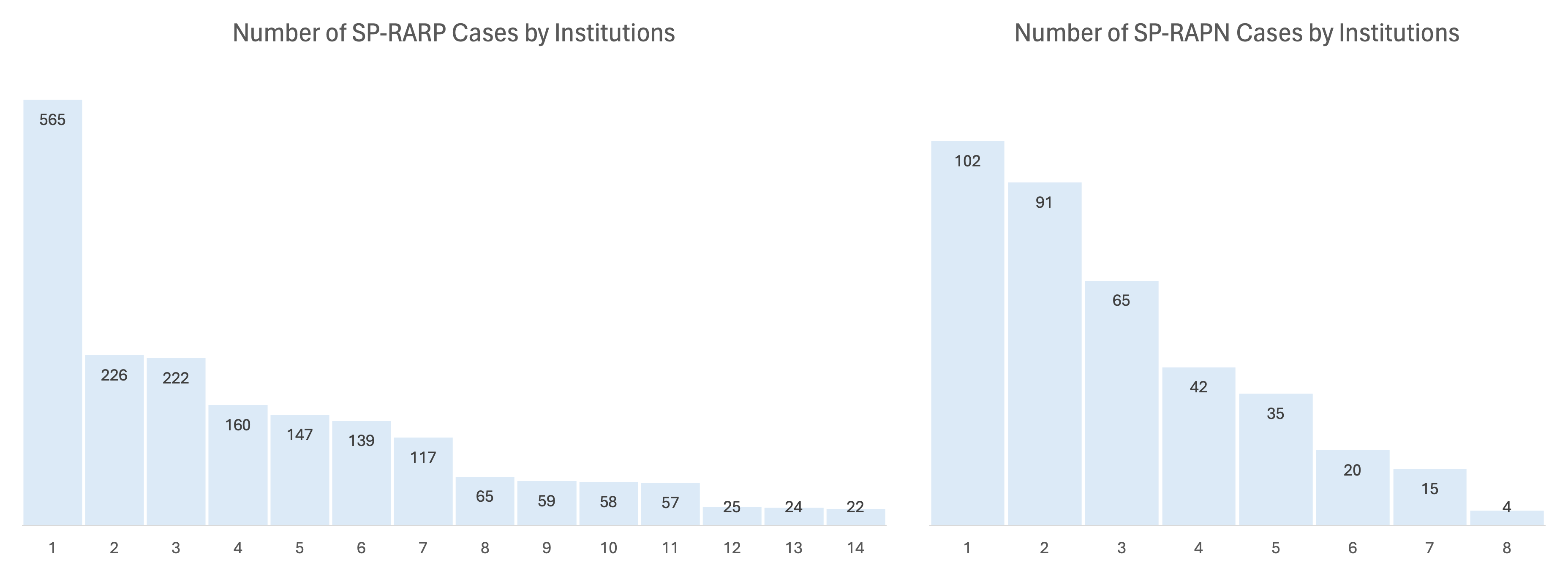

Supplement: Supplementary file 1 — Supplementary file1 (PNG 153 KB) Fig. S1. Case contributions from participating institutions of the Single Port Advanced Research Consortium [file 11701_2025_2796_MOESM1_ESM.png]
